# Obituary—Dr. Slack

**Published:** 1866-08

**Authors:** 


### DEATH OF DR. SLACK.

DIED—On the 29th of May last, Rev. ELIJAH SLACK, M. D., L.L. D., after an illness of several months' duration. We should have noticed this earlier, but it was overlooked.

Dr. Slack was a native of Bucks County, Pennsylvania; was born November 6th, 1784, and was, therefore, in his 82d year when he died. He graduated at Princeton in 1810, and, for a time, conducted an Academy at Trenton. He was afterward appointed Professor of Natural Science and Vice-President of Princeton College. In 1817 he came to Cincinnati, and was a member of the Faculty of Cincinnati College during the existance of that institution. In 1819 the Medical College of Ohio was organized; and he became Professor of Chemistry, holding the position for fourteen years, associated with Eberle, Drake, etc. In 1846 he was appointed Lecturer on Chemistry in the Ohio College of Dental Surgery, and it is supposed delivered the first systematic course of lectures on chemistry as applied to dentistry ever listened to.

Dr. Slack was an ordained minister when he came west, and remained a member of the Presbytery of Cincinnati while he lived. Age and bodily infirmity have kept him from active duties for several years; and he died as a machine runs down, leaving an aged widow and several children to rejoice that he has gone before. For, having fought the good fight, finished his course, and kept the faith, why should not the veteran soldier enjoy his triumph in that peace which passeth all understanding?

